# Relationship between HALP and PNI score with 1-month mortality after CABG

**DOI:** 10.3389/fnut.2024.1489301

**Published:** 2024-11-01

**Authors:** Ilhan Koyuncu, Emin Koyun

**Affiliations:** ^1^IDepartment of cardiology, Izmir Bakircay University, Izmir, Türkiye; ^2^Sivas Numune Hospital, Sivas, Türkiye

**Keywords:** nutrition, coronary artery bypass grafting, mortality, preoperative scores, coronary heart disease

## Abstract

**Background:**

Coronary heart disease (CHD) is the most common cause of cardiovascular disease (CVD). CHD is among the most common causes of mortality and morbidity world wide. In addition, CHD is one of the most important causes of health expenditures world wide. Today, coronary artery bypass grafting (CABG) operations are a widely used surgical procedure and have an important place in the treatment of CHD. Many scoring systems have been evaluated to estimate the risk of mortality and morbidity. 30-day mortality rates after CABG have been reported as 1–4% in large-scale studies.

**Objectives:**

The aim of our study was to evaluate the relationship between 1-month mortality in patients undergoing CABG and the Hemoglobin, albumin, lymphocyte, platelet index (HALP score) and Prognostic nutritional index (PNI) calculated using laboratory data in the preoperative period.

**Methods and design:**

A total of 239 patients who underwent CABG were evaluated retrospectively. Preoperative biochemical and hemogram values, demographic characteristics, comorbidities, HALP score and PNI values of the patients were recorded. The patients were divided into two groups: Exitus group (*n* = 51) and survival group (*n* = 188). The data of the two groups were compared, mainly HALP score and PNI.

**Results:**

It was observed that 51 of 239 patients (21.3%) developed exitus during the 30-day follow-up after CABG. When demographic data are compared; advanced age, female gender, history of DM (Diabetes Mellitus), history of HL (hyperlipidemia) and smoking were found to be associated with mortality. When laboratory data are compared; high troponin levels, low hemoglobin, low lymphocyte and low albumin levels were found to be associated with mortality. Low HALP score (*p* < 0.001) and low PNI (*p* < 0.001) were also found to be associated with mortality. In univariate and multivariate regression analysis; advanced age, history of DM, HALP score and PNI were found to be independent predictors of 30-day mortality after CABG. It was determined that a cut-off value of 0.29 for the HALP score and 39.1 for PNI had found, respectively, 81 and 79% sensitivity and 82 and 80% specificity.

**Conclusion:**

Preoperatively measured HALP score and PNI can be used to predict 1-month mortality after CABG.

## Introduction

Coronary heart disease (CHD) is the most common cause of cardiovascular disease (CVD). CHD is among the most common causes of mortality and morbidity world wide (1, 2). In addition, CHD is one of the most important causes of health expenditures world wide. Today, coronary artery bypass grafting (CABG) operations are a widely used surgical procedure and have an important place in the treatment of CHD. Mortality rates after CABG appear as an important indicator to evaluate the quality of healthcare services provided to patients (3). Many scoring systems have been evaluated to estimate the risk of mortality and morbidity. 30-day mortality rates after CABG have been reported as 1–4% in large-scale studies (4). According to STS (Society of Thoracic Surgeon), mortality rates were reported as 2.3% for CABG and 3.4% for valve surgery (5).

In recent years hemoglobin, albumin, lymphocyte, platelet index (HALP), calculated by the formula Hemoglobin (g/dL) × Albumin (g/dL) × Lymphocyte 103/uL/Platelet 103/uL, has been used to determine the prognosis of multiple tumors, heart failure (HR) and stroke (6–9). However, there is no data regarding the HALP score and mortality after CHD and CABG. Prognostic nutritional index (PNI) (calculated as serum albumin (g/dL) level × 10) + (lymphocyte (10^3^/uL) × 0.005) is used to obtain prognostic information in patients with malignancy, heart failure, peripheral artery disease (PAD), stroke and coronary artery disease (10). Many previous studies have shown an association of PNI with the prevalence of previous CHD. In patients previously had transient ischemic attack or ischemic stroke and received thrombolytic therapy for acute cerebrovascular attack was observed to be higher in the low PNI group than in the high PNI group (9). It has been shown that there is a relationship between the prevalence of multivessel disease and low PNI in patients with coronary artery disease (10). Many observational studies have evaluated the clinical outcomes of PNI in various diseases. Information regarding the effectiveness of PNI in predicting clinical outcomes is limited and contradictory.

There is an increasing amount of research in the literature on the CHD mortality of HALP score and PNI. In recent studies conducted in patients with CHD, there are suggestions that the use of HALP score and PNI in determining mortality rates can provide more comprehensive information about the health status of patients (11, 12).

## Objectives

Determining the power of HALP score and PNI in predicting mortality after CABG; may guide clinicians in improving surgical outcomes. In light of this information, the aim of this study is to examine the relationship between HALP score and PNI with 1-month mortality after CABG and to evaluate the impact of these clinical scoring systems on surgical outcomes.

## Materials and methods

### Patient population

Our study was designed retrospectively. Patients who had CABG between January 2010 and January 2024 were included in the study. Patients were followed for 30 days after CABG. Afterwards, the patients were divided into two groups: those who died and those who survived. Biochemical parameters, demographic characteristics, HALP score, PNI score and comorbidities were compared between both groups. Criteria for inclusion in the study are as follows: having undergone CABG, the patient having a 30-day follow-up after CABG, and being over 18–85 years of age. Exclusion criteria are as follows: missing follow-up data, severe chronic kidney failure, severe liver failure, malignancy, infection, severe valvular heart disease, HFrEF (heart failure with reduced ejection fraction) and deficiencies in blood parameters. This study was conducted according to the guidelines laid down in the Declaration of Helsinki and all procedures involving human subjects/patients were approved by the [Bakırcay University non-invasive ethics committee; ethics number: 1436]. Since the study was retrospective, written or verbal consent was not obtained from the patients.

### Defining HALP score and PNI

PNI score was calculated with (serum albumin (g/dL) level × 10) + (lymphocyte (10^3^/uL) × 0.005) formula.

HALP score was calculated with Hemoglobin (g/dL) × Albumin (g/dL) × Lymphocyte 10^3^/uL/Platelet 10^3^/uL formula.

### Statistical analysis

The Kolmogorov–Smirnov test was used to determine the normal distribution of the data. Mann–Whitney U test or unpaired Student T test was used to detect difference in continuous variable data. Continuous data were expressed as mean ± standard deviation (SD) or median and interquartile range median (minimum–maximum). Categorical variables were expressed as absolute (*n*) and relative frequencies (%) and as percentage (%*n*). Chi square analysis was used to compare categorical variables. Univariate and multivariate Cox regression analysis was performed to determine parameters predicting mortality. Variables that were statistically significant (*p* < 0.05) in univariate Cox regression analysis were included in multivariate Cox regression analysis. Receiver operating characteristic (ROC) curve analysis was performed to obtain the cut-off value and area under the curve (AUC) of parameters predicting mortality.

## Results

A total of 239 patients who underwent CABG between 2010 and 2024 were included in the study. Preoperative characteristics and laboratory values of patients who did and did not develop mortality were compared (Table 1). When demographic data are compared; older age (*p* < 0.001), female gender (*p* = 0.006), having a history of DM (*p* < 0.001), having a history of HL (*p* = 0.009) and smoking (*p* = 0.02) were found to be associated with mortality. When laboratory data are compared; high troponin (*p* = 0.002) low hemoglobin (*p* < 0.001), low lymphocyte (*p* < 0.001) and low albumin (*p* < 0.001) levels were found to be associated with mortality. Low HALP score (*p* < 0.001) and low PNI (*p* < 0.001) were also found to be associated with mortality. There was no difference between the two groups in the echocardiographic evaluation of the patients in the preoperative period (Table 2).

**Table 1:** Comparison of variables between both groups.

Variables	Ex. patients (*n*: 51)	Survived patients (*n*: 188)	*p*
Age (years)	69.5 ± 7.8	62.7 ± 9.2	<0.001
Female, *n* (%)	21 (41.1)	47 (25)	0.006
BMI	26.9 ± 2.9	26.7 ± 3.5	0.74
DM, *n* (%)	20 (39.2)	45 (23.9)	<0.001
HT, *n* (%)	33 (64.7)	135 (71.8)	0.99
HL, *n* (%)	31 (60.8)	68 (36.1)	0.009
Smokers, *n* (%)	13 (25.5)	34 (18)	0.02
Systolic BP (mmHg)	131 ± 26	136 ± 19	0.61
Diastolic BP (mmHg)	78.8 ± 11	78.9 ± 9	0.71
CABG vessel	3.1 ± 0.9	2.9 ± 0.8	0.42
Emergency CABG, *n* (%)	12 (23.5)	35 (18.6)	0.06
Emergency PCI, *n* (%)	8 (15.7)	22 (11.7)	0.29
Creatinine (mg/dL)	1.13 ± 0.4	1.07 ± 0.3	0.63
ALT (IU/L)	21.3 ± 3.1	19.8 ± 2.9	0.34
AST (IU/L)	16.7 ± 3.9	21.2 ± 3.2	0.07
GGT (IU/L)	25 ± 7.1	31 ± 9.3	0.37
Cholesterol (mg/dL)	180.7 ± 48	194.6 ± 41	0.08
HDL (mg/dL)	45.7 ± 9.2	42.5 ± 8.8	0.41
LDL (mg/dL)	122.8 ± 49	128.7 ± 40	0.65
Troponin	14.1 ± 3.2	7.4 ± 1.9	0.002
CRP (mg/dL)	65.4 ± 17	64.2 ± 16	0.96
WBC (10^3^/uL)	11.7 ± 5	10.1 ± 3.1	0.08
HB (g/dL)	10.7 ± 1.4	12.3 ± 2.2	<0.001
Neutrophil (%)	8.9 ± 1.7	8.8 ± 2.1	0.94
Platelet(10^3^/uL)	263.6 ± 27.2	254.7 ± 31.2	0.61
Lymphocyte (%)	1.4 ± 0.7	1.9 ± 0.8	<0.001
Albumin (g/dL)	3.5 ± 0.5	4.1 ± 0.4	<0.001
PNI	35.7 ± 5.2	39.8 ± 4.1	<0.001
HALP	0.31 (0.19–0.52)	0.52 (0.23–0.55)	<0.001

Data are expressed as *n* (%), mean ± standard deviation, median (1st quartile–3rd quartile).

BMI, Body Mass Index; DM, Diabetes Mellitus; HT, Hypertension; HL, Hyperlipidemia; BP, Blood Pressure; CABG, Coronary Artery Bypass Greft; PCI, Percutaneous Coronary Intervention; ALT, Alanine Aminotransferase; AST, Aspartate Aminotransferase; GGT, Gamma Glutamyl Transferase; HDL, High Density Lipoprotein; LDL, Low Dansity Lipoprotein; CRP, C-reactive protein; WBC, White Blood Cell; HB, Hemoglobin; PNI, Prognostic Nutritional Index; HALP, Hemoglobin, Albumin, Lymphocyte, Platelet Index.

**Table 2:** Echocardiographic findings of patient groups.

	Exitus patients (*n*: 51)	Survived patients (*n*: 188)	*p*
LVEF (%)	53.4 ± 7.6	51.5 ± 8.1	0.18
Left atrium diameter (mm)	44.2 ± 4.1	47.2 ± 5.2	0.09
LVSWT (mm)	10.3 ± 1.3	9.9 ± 2.1	0.51
PWT (mm)	9.2 ± 1.5	9.6 ± 1.8	0.89
LVEDD (mm)	48.9 ± 4.1	51.1 ± 3.9	0.13
LVESD (mm)	33.9 ± 3.4	33.4 ± 3.5	0.57

LVEF, Left Ventricular Ejection Fraction; LVSWT, Left Ventricular Septal Wall Thickness; PWT, Posterior Wall Thickness; LVEDD, Left Ventricular End-Diastolic Diameter; LVESD, Left Ventricular End-Systolic Diameter.

In univariate and multivariate regression analysis (Table 3); advanced age (*p* = 0.01), having a history of DM (*p* = 0.01), HALP score (*p* < 0.001) and PNI (*p* < 0.001) were determined as independent predictors of mortality.

**Table 3:** Univariate and multivariate cox regression analysis to find parameters predicting mortality.

Variables	Univariate	*p*	Multivariate	*p*
	Odds ratio (%95 CI)		Odds ratio (%95 CI)	
Age	0.912 (0.713–0.993)	<0.001	0.818 (0.791–0.997)	0.01
Female	0.811 (0.801–0.923)	0.03		
DM	1.022 (1.013–1.135)	<0.001	1.121 (1.098–1.327)	0.01
HL	0.657 (0.541–0.889)	0.01		
Smoker	1.324 (1.211–1.657)	0.04		
Troponin	0.718 (0.699–0.937)	0.02		
PNI	0.587 (0.322–0.889)	<0.001	0.643 (0.588–0.927)	<0.001
HALP	0.632 (0.513–0.916)	<0.001	0.711 (0.629–0.938)	<0.001

CI, Confidence Interval; DM, Diabetes Mellitus; HL, Hyperlipidemia; PNI, Prognostic Nutritional Index; HALP, Hemoglobin, Albumin, Lymphocyte, Platelet Index.

**Figure 1:**
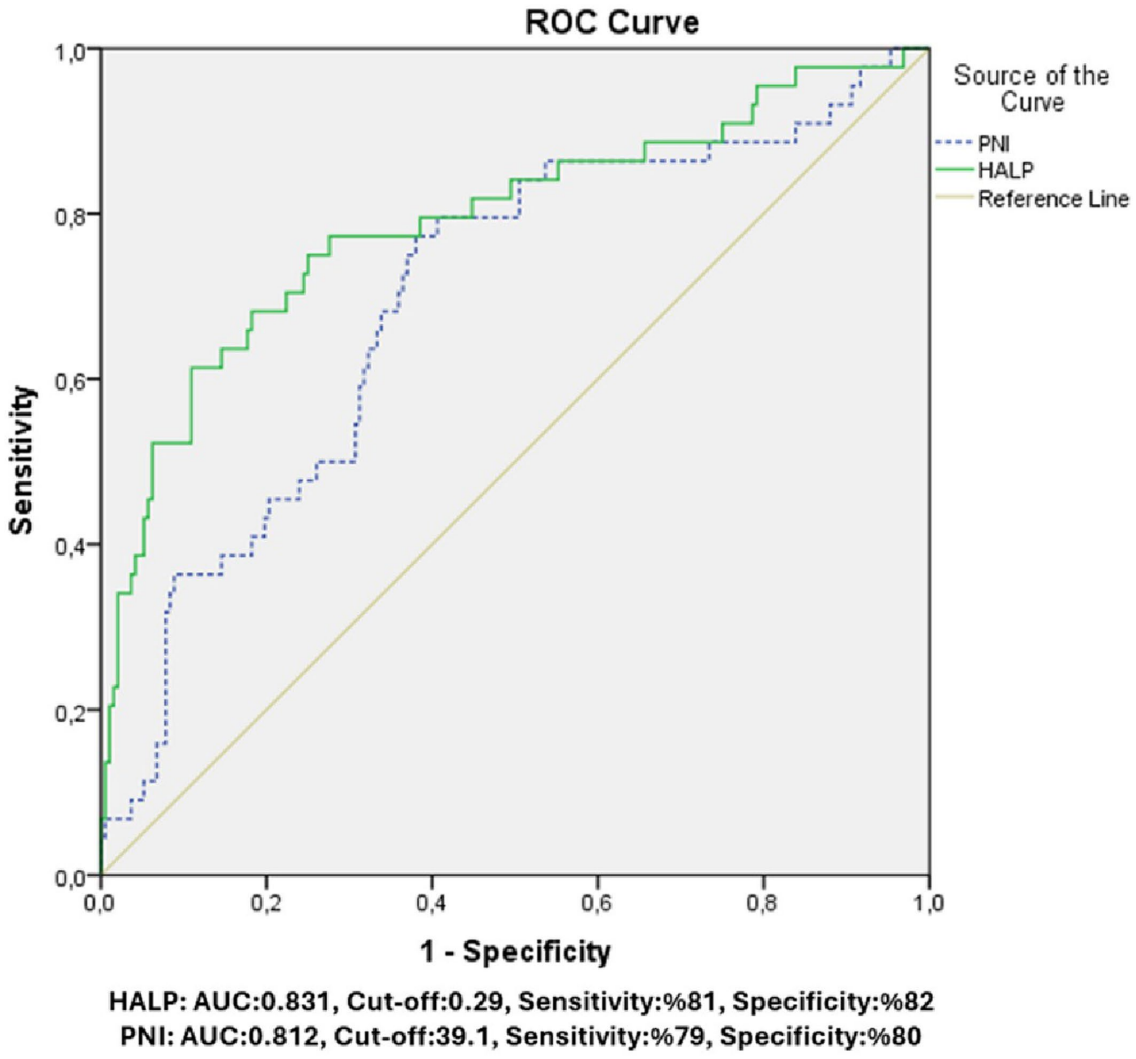
It was determined that a cut-off value of 0.29 for the HALP score had 81% sensitivity and 82% specificity. A cut-off value of 39.1 for PNI was found to have 79% sensitivity and 80% specificity.

In Roc curve analysis (Figure 1) it was determined that cut-off value of 0.29 for the HALP score had 81% sensitivity and 82% specificity, cut-off value of 39.1 for PNI was found to have 79% sensitivity and 80% specificity for predicting mortality.

## Discussion

In this study, we evaluated the relationship between HALP score and PNI with in-hospital 30-day mortality after CABG. Data on simple scoring systems for complications and mortality after CABG were previously available in the literature (13–16). Our results showed that age, history of DM, HALP score, and PNI were independent predictors of 1-month mortality after CABG. Our findings, like the current literature, showed that increasing age and the presence of a history of DM were associated with increased mortality (17–19). In this study, the mortality rate after CABG was found to be higher than the current literature. This was attributed to the inclusion of patients who underwent emergency surgery in the study.

It is very difficult to identify predictors associated with outcomes after CABG. Assessment of each patient’s personal characteristics and the severity of the disease is quite complex and difficult. For this purpose, the EuroSCORE system is one of the most commonly used scoring system and is quite specific for cardiac surgery. EuroSCORE system is a scoring system in which patient’s age, gender and many comorbidities (renal failure, pulmonary diseases, peripheral artery disease, endocarditis and DM) are evaluated.

Anemia is very important in the clinical outcome of CHD (20–22). In a previous study; low hemoglobin (HB) had shown to be an independent predictor of cardiovascular events and mortality in patients with stable CHD (23). Albumin is an indicator of nutritional status. The prognostic value of in patients with CHD has been evaluated in many studies (24). In a study (25), it was found that the serum albumin level measured before percutaneous coronary interventions (PCI) may be among the major adverse cardiac events in patients with CHD. Additionally, the status of the inflammatory system is also an important factor affecting the prognosis of CHD. Both acute inflammatory response and low lymphocyte count have been found to be strongly associated with poor prognosis in CHD (26). The role of platelets in the acute and chronic inflammatory process of CHD is well known (27). In one study, platelet count was found to be associated with increased mortality due to CHD in postmenopausal women (28). The interaction between anemia, malnutrition and inflammation may affect the prognosis of the disease (29). Albumin level decreases due to low synthesis during inflammation (30). Excessive stockin production during inflammation restricts iron uptake. For this reason, HB production decreases and as a result, anemia occurs (31). Additionally, nutritional deficiency occurring in CHD patients also causes anemia. In addition, nutritional status and inflammatory response disorders of patients can be observed after CABG (32). Myocardial fibrosis-strain-hypertrophy-dysfunction, myocyte death and apoptosis, kidney damage, hemodynamic stress, renin-angiotensin-aldosterone system (RAS) activation, oxidative stress, inflammation, angiogenesis and vascular cell proliferation have been shown to be important mechanisms of CVD mortality (1). In addition to these conditions, nutritional status also has a primary role in cardiovascular functions. Low or high BMI (body mass index) is associated with cardiac metabolism and functions in CHD and HF (heart failure) patients (33).

The HALP score is a combination that includes hemoglobin, albumin, lymphocyte and platelet values. It is used to evaluate inflammation and nutritional status and to predict clinical outcomes for many diseases, especially malignancies (6, 8, 9, 34). There are not enough studies examining the prognostic value of HALP score in CHD. However, in a study conducted on patients with acute ischemic stroke; it was found that low HALP score was associated with increased 90-day and 1-year mortality (23). In another study conducted in patients with CHD, low HALP score was found to be associated with increased mortality (29). In our study, we found that low HALP score was associated with increased mortality, consistent with previous literature. Additionally, in univariate and multivariate regression analysis, advanced age was determined to be an independent predictor of 1-month mortality after CABG, as were DM and PNI.

PNI is an index that reflects nutritional status and evaluates immune system function. Today, many nutritional indices have been defined. These are Subjective Global Assessment (SGA) (35), Mini Nutrition Assessment (MNA) (36), Malnutrition Inflammation Score (MIS) (37), Geriatric Nutrition Risk Index (GNRI) (38), Controlled Nutrition Status (CONUT) score (39) and Prognostic Nutrition Index (PNI) (40). SGA, MNA and MIS are evaluated by experienced clinicians according to the clinical conditions of the patients and are subjective evaluations. Nutritional indices such as GNRI, CONUT score and PNI are objective indices calculated using widely used and inexpensive markers. GNRI is calculated using serum albumin level and body mass index (BMI). Nutritional indices such as CONUT score and PNI have become more frequently used nutritional indices today. These two indices are easy and practical to calculate, as they are calculated with routinely used laboratory parameters. The CONUT score is measured using laboratory tests such as serum albumin concentration, total lymphocyte count, and total cholesterol concentration. The CONUT score is widely used as a screening tool for the early detection of malnutrition (39). PNI is calculated with the formula 10 × serum albumin (g/dL) + 0.005 × lymphocyte count (/μL). Serum albumin is used as an indicator of protein reserve and total cholesterol is used as an indicator of energy depletion. The total lymphocyte count provides information about the status of the immune system (39). Therefore, CONUT score and PNI not only provide information about nutritional status, but also provide information about the immune system. There are currently a limited number of studies in the literature regarding mortality after CABG, but similar results were obtained in these studies. For example, a study in patients with coronary artery disease reported that low PNI was associated with long-term clinical outcomes (25). In our study, we found that low PNI was associated with increased mortality, consistent with previous literature. Additionally, in univariate and multivariate regression analysis, advanced age was determined to be an independent predictor of 1-month mortality after CABG, as were DM and HALP score.

In this study, we also found significant relationship between HALP score and PNI with 30-day mortality after CABG. It was determined that patients with lower HALP scores had a higher risk of mortality. Similarly, patients with low PNI were also associated with worse surgical outcomes. In our study, we also investigated the potential predictive values of HALP score and PNI. HALP score below 0.29 and PNI below 39.1 were found to be associated with an increased risk of mortality.

## Conclusion

This study has some limitations. Due to its retrospective design, there may be omissions or errors in the data collection process. Additionally, only a single center’s data were used, the results may have limitations in terms of generalizability. Moreover, nutritional indices such as HALP score and PNI are still areas of active research and need further study. Prospective and multicenter studies are needed to examine the impact of these indices on surgical outcomes in more detail.

In conclusion, clinical scoring systems such as HALP score and PNI may be potentially useful tools in assessing in-hospital mortality after CABG. However, further research is needed on how widespread the use of these indices is in routine clinical practice and how proven their effectiveness is.

## Data Availability

The data analyzed in this study is subject to the following licenses/restrictions: hb, alb, plt. Requests to access these datasets should be directed to Ilhan Koyuncu, dr_ilhann@hotmail.com.
